# Cancer in the Spine: Comprehensive Care

**DOI:** 10.1038/sj.bjc.6603380

**Published:** 2006-11-28

**Authors:** J P Dormans, G Mik, O Hamer

**Affiliations:** 1 University of Pennsylvania School of Medicine and The Children's Hospital of Philadelphia, Philadelphia, PA, USA

*Cancer in the Spine* is one of the flagship books in the ‘Current Clinical Oncology’ series. Basic principles and recent concepts in the diagnosis, staging, and treatment of spinal tumours are highlighted for physicians, including internists, oncologists, neurosurgeons, and orthopaedic surgeons. Concepts in chemo- and radiation therapy, pain management, and reconstructive surgery and palliative care of the primary and metastatic spinal tumours are also emphasised. In a single volume, the authors present a contemporary vision from various disciplines to provide a guide to multidisciplinary management of all details of spinal tumours. There are 43 chapters, 379 pages, from 65 contributors or coauthors who cover all concerns of spinal tumours. The editor, Robert F McLain is the director of the Spine Research program of the Cleveland Clinic Foundation and most of the section editors are based at the Cleveland Clinic.

The first 12 chapters provide a useful introduction containing up-to-date summaries of incidence and demographics (with detailed tables), pathophysiology, characteristics of tumours, methods of screening and diagnosis, and principals of treatment. The chapter on metastatic disease provides information regarding the molecular biology of metastases and angiogenesis as well as cell adhesion and invasion. In chapters 13–20, common primary cancers such as multiple myeloma, metastatic breast, lung, thyroid, and prostate, which have affinity to metastasize to the spine, are discussed. These sections provide a good point of view and update of recent progress for orthopaedists and neurosurgeons who are not familiar with these branches. There is also a chapter including carcinoma of unknown primary, which describes easy to follow algorithms for evaluation. Chapters 22–25 concentrate on spinal radiotherapy and contain useful information updates for orthopaedists and neurosurgeons on how radiation is currently prescribed and administered. These chapters discuss concepts of technique, radiotherapy of the paediatric patients, conventional doses and treatment schedules, and photon and proton beam radiotherapy, with clear illustrations and recent references. In the following section there are 12 chapters in which the authors present different types of surgical techniques, comprehensive steps of surgical management including patient selection and staging for surgery, surgical complications, and general goals for surgery. Surgical techniques and approaches are discussed in detail, from biopsies to more complicated resections and reconstructions are demonstrated with quality pre- and postoperative images, diagrams, and illustrations. There are quality illustrations of procedures such as lumbosacral resection and reconstruction. In the last five chapters, bracing, rehabilitation, pain management, and screening are discussed. These chapters are very helpful; the use of tables in the section of rehabilitation and bracing provides useful summaries. Sections on palliative care and pain management are included. The last chapter deals with quality of life, cost effectiveness, patients' fears and expectations, and surgeons' expectations. It is unusual to find topics like these in current textbooks.

Spinal tumours are rare and a vast majority of the spinal tumours that we encounter are metastatic. While there are several books on spines, there are but a few that focus on this specific topic. *Cancer in the Spine* is a comprehensive and a unique text that clearly meets the purpose of comprehensive care in spinal cancers and provides excellent resources. Chapters are generally supported by impressive current reference lists which are an invaluable resource for clinical and research practices. It deserves a place in the library of any specialist or institution that is involved in the care of individuals with cancer involving the spine.

